# Tackling Soil ARG‐Carrying Pathogens with Global‐Scale Metagenomics

**DOI:** 10.1002/advs.202301980

**Published:** 2023-07-09

**Authors:** Binhao Wang, Jianming Xu, Yiling Wang, Erinne Stirling, Kankan Zhao, Caiyu Lu, Xiangfeng Tan, Dedong Kong, Qingyun Yan, Zhili He, Yunjie Ruan, Bin Ma

**Affiliations:** ^1^ Zhejiang Provincial Key Laboratory of Agricultural Resources and Environment Institute of Soil and Water Resources and Environmental Science College of Environmental and Resource Sciences Zhejiang University Hangzhou 310058 P. R. China; ^2^ Hangzhou Global Scientific and Technological Innovation Center Zhejiang University Hangzhou 310058 P. R. China; ^3^ Agriculture and Food Commonwealth Scientific and Industrial Research Organization Adelaide 5064 Australia; ^4^ School of Biological Sciences The University of Adelaide Adelaide 5005 Australia; ^5^ Institute of Digital Agriculture Zhejiang Academy of Agricultural Sciences Hangzhou 310021 P. R. China; ^6^ Xianghu Laboratory Hangzhou Zhejiang 311200 P. R. China; ^7^ Southern Marine Science and Engineering Guangdong Laboratory (Zhuhai) Zhuhai 519080 P. R. China; ^8^ Institute of Agricultural Bio‐Environmental Engineering College of Bio‐Systems Engineering and Food Science Zhejiang University Hangzhou 310058 P. R. China; ^9^ The Rural Development Academy Zhejiang University Hangzhou 310058 P. R. China

**Keywords:** ARG‐carrying pathogens, biogeography, diversity, global scale, soil

## Abstract

Antibiotic overuse and the subsequent environmental contamination of residual antibiotics poses a public health crisis via an acceleration in the spread of antibiotic resistance genes (ARGs) through horizontal gene transfer. Although the occurrence, distribution, and driving factors of ARGs in soils have been widely investigated, little is known about the antibiotic resistance of soilborne pathogens at a global scale. To explore this gap, contigs from 1643 globally sourced metagnomes are assembled, yielding 407 ARG‐carrying pathogens (APs) with at least one ARG; APs are detected in 1443 samples (sample detection rate of 87.8%). The richness of APs is greater in agricultural soils (with a median of 20) than in non‐agricultural ecosystems. Agricultural soils possess a high prevalence of clinical APs affiliated with *Escherichia, Enterobacter, Streptococcus*, and *Enterococcus*. The APs detected in agricultural soils tend to coexist with multidrug resistance genes and *bacA*. A global map of soil AP richness is generated, where anthropogenic and climatic factors explained AP hot spots in East Asia, South Asia, and the eastern United States. The results herein advance this understanding of the global distribution of soil APs and determine regions prioritized to control soilborne APs worldwide.

## Graphical abstract

The ultimate risk of antibiotic abuse is the spread of antibiotic resistance genes (ARGs) into pathogens. This work reveals that soilborne AP diversity is driven by human activities, climatic variables, and physicochemical properties at the global scale. Specifically, agricultural land has the highest AP richness. AP hotspots are located across East Asia, South Asia, and the eastern United States.

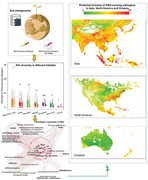

## 1 Introduction

The increase in plant, animal, and human pathogens is resulting in negative outcomes such as food yield losses and public health crises.^[^ ^1^, ^2^ ^]^ One issue further compounding pathogen pressures is the prevalence of antibiotic resistance genes (ARGs) in the environment that have emerged due to widespread antibiotic use.^[^ ^3^ ^]^ This selection pressure has promoted the mobilization of a large range of ARGs to many microorganisms, particularly to those causing disease.^[^ ^4^ ^]^ As a consequence, there are increasing difficulties in preventing and treating infections caused by ARG‐carrying pathogens (APs), which pose threats to global health^[^ ^5^, ^6^ ^]^ and economic development.^[^ ^7^, ^8^, ^9^, ^10^ ^]^ The extreme consequences of which could cause an additional 10 million deaths per year and a loss of up to US$100 trillion from the global GDP by 2050.^[^ ^11^ ^]^

In recent years, interest in the biogeography of the soil antibiotic resistome on a global scale has dramatically increased. Delgado‐Baquerizo et al.^[^ ^12^ ^]^ reported that ARG proportion peaked in high latitude cold and boreal forests relative to other global environments with climatic seasonality being a key driver of their global distribution. Zheng et al.^[^ ^13^ ^]^ showed ARG abundance in agricultural habitats was higher than that in non‐agricultural habitats, and that agricultural activities and anthropogenic factors explained global ARG hotspots. Despite reports on the occurrence, distribution, and drivers of soil antibiotic resistome at global scales, we still lack a comprehensive understanding of the distribution of APs at a broad scale.^[^ ^14^ ^]^ ARG‐carrying pathogens are considered the ultimate risk of antibiotic resistance and result from the spread of ARGs into pathogens. However, only a few APs have been studied in sufficient detail to gain insights into the global determinants of their distribution.^[^ ^15^, ^16^ ^]^ Therefore, a comprehensive study of soil APs across habitat types is needed to identify factors influencing the diversity of soilborne APs at a global scale. This information is essential to identify global soil AP hotspots. Such information is crucial to focus on early surveillance, prevention, and intervention of APs, which can guide management actions aimed to reduce drug‐resistant pathogen infections.^[^ ^17^ ^]^

To address existing knowledge gaps related to soilborne AP distribution and drivers, we characterized them using 1643 deep‐sequenced soil metagenomes collected from across the globe. This global soil metagenome dataset allowed us to answer three main questions. First, what is the overall richness of soilborne APs in different habitats? We expected the richness to vary greatly by habitat. Agricultural lands severely affected by human activities are rich in pathogens and ARGs, while habitats such as tundra are restricted to a few pathogens and ARGs. Second, what are the occurrence and characteristics of antibiotic resistance genes within APs? While there is some speculation that the resistome may not be related to microbial taxonomic composition due to resistome mobility,^[^ ^18^ ^]^ other studies have shown high interpretability for total variations of the resistome composition by taxonomic composition.^[^ ^19^, ^20^ ^]^ Finally, what drives variation in soil AP richness at a global scale? We predicted that anthropogenic and climatic factors—previously identified as important drivers of soil ARG biogeography^[^ ^13^ ^]^—would also influence AP diversity variation.

## 2 Results

### 2.1 Identification of soil APs at a Global Scale

**Figure 1:**
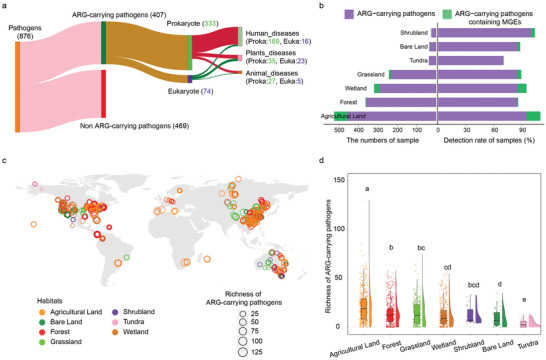
Annotation overview of soil ARG‐carrying pathogens (APs) at the global scale. a) Overall characteristics of ARGs carriers, taxonomy and pathogenicity of annotation pathogens based on the constructed database. “Proka” indicates “prokaryote” and “Euka” indicates “eukaryote”. b) The number and detection rate of samples with APs and APs containing mobile genetic elements (MGEs). c) Global profile of AP richness for each sample. A total of 1643 samples were collected and filtered to the 1443 samples containing APs. d) Comparison of AP richness (*n* = 1443) in seven habitats. Each sample is represented by a dot with horizontal jitter for visibility. The horizontal box lines represent the first quartile, the median, and the third quartile. Boxplot midline indicates data median. Different letters indicate statistically significant difference.

To detect soilborne APs globally, we annotated contigs assembled from high quality reads of 1643 soil metagenomes using pathogens‐DB (including the PHI‐base and T16 National archives) and ARGs‐DB (including SARG v2.2) simultaneously (Figure S1, Supporting Information and Supporting Data 1–2). A total of 876 pathogens (pathogen sequence proportion ranging from <0.001% to 6.7%, average of 0.5%) and 24 ARG types (ARG abundance varying among habitats, ranging from 0.21 to 0.51 resistance genes per cell, average of 0.31) were detected in the soil samples (Figure S2, Supporting Information). We identified 407 APs, including 333 prokaryotic APs and 74 eukaryotic APs, from 1443 soil metagenomes (87.8% of all 1643 soil metagenomes) (**Figure** **1**a). The relative abundance of APs was low, with an average of 0.003% (Figure S3a,b, Supporting Information), while the occurrence of APs in seven habitats was high, ranging between 70% and 100% (Figure 1b). Of the detected APs, 90 APs carrying mobile gene elements (MGEs) (22.1% of all 407 APs) were identified from only 111 soil metagenome samples (6.8% of the total 1643 soil metagenome samples) (Figure 1b). The richness of detected APs was highest in agricultural soils (median of 20) and lowest in tundra soils (median of 4) (**Figure** **3**c,d). All 1015 genes (ARG subtypes) conferring almost all major classes of antibiotics such as vancomycin, bacitracin, tetracycline, and macrolide‐lincosamide‐streptogramin, were detected within APs (Figure S3c,d, Supporting Information and Supporting Data 3). The detected prokaryotic APs were assigned to infect hosts (the dataset covers species associated with experimentally verified and predicted pathogens), including 169 human pathogens, 35 plant pathogens, and 27 animal pathogens. Meanwhile, the detected eukaryotic APs were assigned infecting hosts including 16 human pathogens, 23 plant pathogens, and 5 animal pathogens (Figure 1a).

### 2.2 Taxonomic Profile of APs in Soils

**Figure 2:**
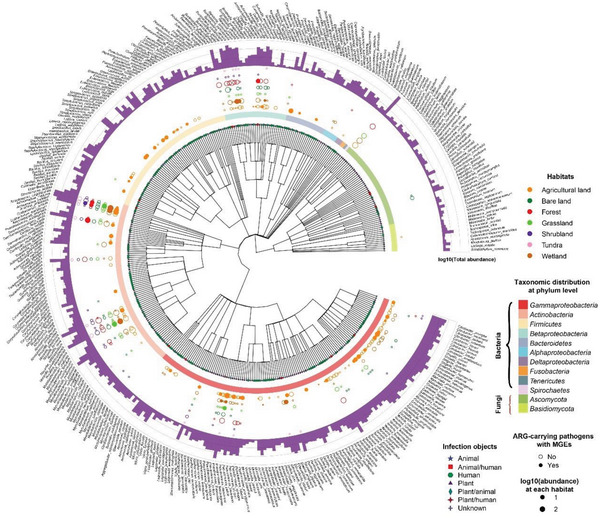
Composition profile of ARG‐carrying pathogens (Bacteria and Fungi) in different habitats distributed globally. Phylogenetic distribution of representative bacterial and fungal APs (Here, Proteobacteria was further divided into Alpha‐, Beta‐, Gamma‐, and Deltaproteobacteria) in the soil metagenomes. The outer circle plots the abundance of APs (displayed as a histogram) in all samples. The middle circle plots the MGE profile of APs. Dot size represents abundance of APs in each habitat and the solid and open circles represent the presence or absence of MGEs within pathogen, respectively. The inner circle indicates major taxa of APs at the phylum level. The different shapes at the ends of the branches represent the infection objects of APs.

We constructed a phylogenetic tree for 388 APs that belonged to 7 bacterial phyla and 2 fungal phyla (**Figure** **2** and Supporting Data 4). We found that most dominant APs occurred in agricultural soils, suggesting that the diversity of APs was greatest in agricultural soils. APs affiliated with *Gammaproteobacteria, Betaproteobacteria*, and *Actinobacteria* were dominant in seven habitats, whereas APs affiliated with *Bacilli* and *Clostridia* (*Firmicutes* phylum) were only dominant in agricultural soils. Notably, human/clinical pathogens, such as *Escherichia*, *Enterobacter, Streptococcus, Enterococcus, Pectobacterium*, *Klebsiella*, *Pseudomonas*, *Nocardia*, and *Mycolicibacterium* were relatively abundant. Of the detected APs, some human pathogens including *Corynebacterium urealyticum*, *Streptococcus suis*, *Pectobacterium carotovorum*, and *Shigella dysenteriae* are unique to agricultural soils. Moreover, the proportion of APs with MGEs was also substantially higher in agricultural soils (14.8%) than those from other habitats (0%–9.5%).

### 2.3 Antibiotic Resistome of APs across Different Habitats

**Figure 3:**
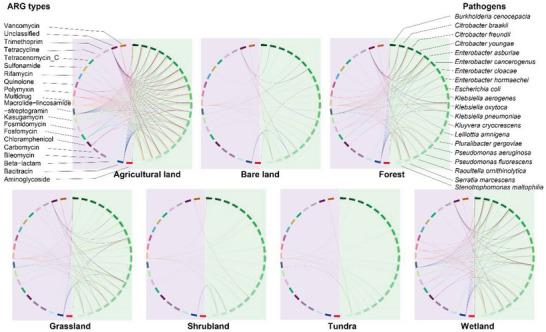
Co‐occurrence patterns among ARG types and their host pathogens in seven habitats. Bipartite network representing interactions from the global soil samples involving pathogen (right ribbons) and types of ARG (left ribbons), in which ribbons connecting two segments indicate copresence links. The edges represent at least one occurrence of the pathogens targeting the corresponding ARGs. The density of connecting lines is proportional to the observed co‐occurrence frequencies between pathogen species and ARG types. ARG color indicates their drug‐resistant type.

We constructed a bipartite network to decipher the overall interconnection between pathogens and ARGs within APs (Figure S4a, Supporting Information). The most common ARG subtypes were multidrug resistance genes and the bacitracin resistance gene *bacA*. Most APs were human pathogens, such as *Enterobacter cloacae*, *Klebsiella pneumoniae*, *Escherichia coli*, and *Enterobacter asburiae*. The APs detected in agricultural soils had the highest number of ARGs, and the highest proportion of APs with MGEs (Figure S4b, Supporting Information). To remove the effect of sampling size, we normalized the number of ARGs per pathogen within each habitat and found that APs in agricultural soils still possessed the highest number of ARGs among the seven habitats (Figure S4b, Supporting Information). We further analyzed the antibiotic resistome of dominant APs in different habitats (Figure 3). Overall, multidrug, fosmidomycin, bacitracin, beta‐lactam, macrolide‐lincosamide‐streptogramin, and kasugamycin were the top six most dominant ARG types, which were typically carried by APs belonging to *Burkholderia*, *Citrobacter*, *Enterobacter*, *Escherichia*, *Klebsiella*, *Pseudomonas*, and *Serratia* genera. Notably, *Enterobacter asburiae*, *Escherichia coli*, *Citrobacter youngae*, and *Citrobacter freundii* carried multiple types of ARGs resistant to more than five classes of antibiotics in agricultural soils, while no ARGs were detected in these pathogens from soils of shrubland and tundra.

### 2.4 Phylogenetic Coherence of the Resistance Profiles of APs

**Figure 4:**
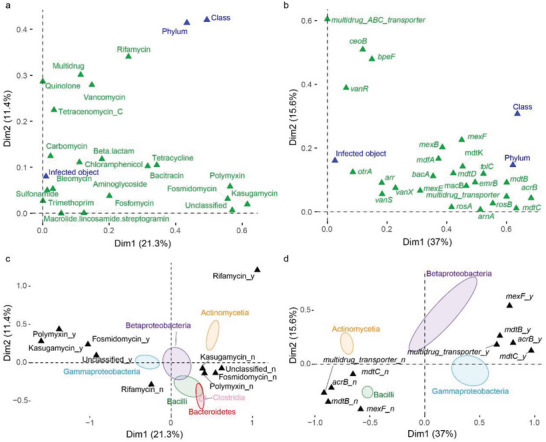
Multiple correspondence analysis (MCA) of antibiotic resistance genes and their potential host pathogens across seven habitats. a) and b) Variable correlation plot of a multiple correspondence analysis (MCA) visualising 20 ARG types and 25 most linked ARG subtypes as active variables in green and three supporting variables infection objects, phylum, and class in blue. c) and d) Individual biplot maps based on MCA of the taxon class as indicated in the coloured legend.

We employed multiple correspondence analysis (MCA) to explore the relationships between ARGs and AP taxonomic group (at the phylum and class level) as well as infection objects. We selected a total of 20 ARG types and the top 25 subtypes based on their presence/absence as active variables for MCA (**Figure** **4** **;** Figure S5, Supporting Information). The MCA model accounted for a total variance of 32.7% and 52.6% for the first and second dimensions, respectively (Figure 4a,b). We found that the first dimension was correlated with ARGs such as kasugamycin, fosmidomycin and polymyxin, as well as subtype genes *mdtC*, *mdtB*, and *acrB*. In contrast, the second dimension was correlated with rifamycin, quinolone, and multidrug, genes *multidrug_ABC_transporter*, *bpeF* and *ceoB* (Figure 4b). These ARG types/subtypes were important for distinguishing APs (Figure S5a,b, Supporting Information). When introducing phylogenetic lineage and infection objects as supplementary variables to the model, we observed a strong correlation of phylogenetic lineages with both dimensions but little to no correlation for infection objects (Figure 4a,b). This indicates phylogenetic coherence of the resistance profiles within APs. A more detailed analysis of observed class variation, based on the explained variance from the MCA analysis, revealed that the related *Actinomycetia* lineages were characterized by a presence of rifamycin ARGs, while *Gammaproteobacteria* lineages were associated with the presence of polymyxin and kasugamycin (Figure 4c). For ARG subtype, more distantly related *Gammaproteobacteria* and *Bacilli* lineages were characterized by a presence/absence of *mexF*, *mdtB*, *mdtC* and *arcB* genes (Figure 4d). We did not observe a clear separation of human pathogens from plant or animal pathogens when analyzing the impact of infection objects on the observed resistance variance (Figure S5c,d, Supporting Information).

### 2.5 Identification of Primary Factors Driving AP Richness

To examine potential mechanisms driving soil AP diversity patterns across the globe, we selected 16 global datasets (Supporting Data 5) to identify key covariates. These covariates were grouped into four themes: anthropogenic activities, climate, soil properties, and vegetation. Using random forest (RF) analysis, we determined the main predictors for soil AP richness (Figure S6, Supporting Information). We found that anthropogenic factors, such as population density and human development index, were most important variables in predicting AP richness, followed by the soil properties and climate. Specifically, population density (49.9%) contributed more than human development index (47.1%). Precipitation was found to be a more significant factor (38.7%) than temperature (32.5%) in explaining the variation in AP richness among the climate variables. Soil pH (35.5%) contributed more than soil organic carbon (31.5%) among the soil properties.

### 2.6 Projection of Soilborne AP Richness at the Globe

**Figure 5:**
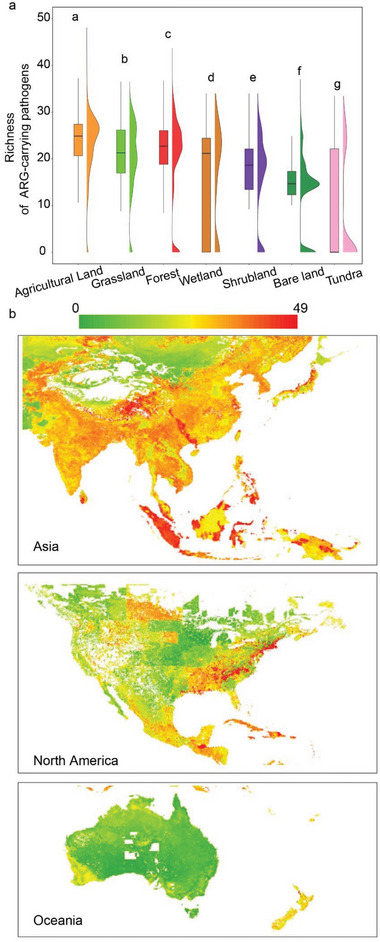
Map of soil ARG‐carrying pathogen (AP) richness. a) Comparison of predicted AP richness in seven habitat types. Different letters indicate statistically significant difference. b) Predicted AP richness in Asia, North America, and Oceania.

Given the crucial role of covariates in driving soilborne AP richness, we attempted to predict AP richness across the globe using the important drivers determined by the RF model. After eliminating the samples that failed to match the environmental parameters, the prediction model for the remaining 1372 soil samples had good performance (R^2^ = 0.77) for richness gradient predictions (Figure S6d, Supporting Information). The final model enabled us to extend this relationship across the global scale to construct an atlas of the AP richness at a 0.01° of resolution (Figure S7, Supporting Information). The prediction accuracy is lowest in arid regions and in parts of the Amazon and Malay Archipelago due to uncertainties caused by uneven and insufficient sampling (Figure S8, Supporting Information). Our results showed that agricultural land generally had the highest AP richness, followed by grassland, forest, wetland, shrubland, bare land and tundra (**Figure** **5**a). To eliminate the issue of insufficient prediction caused by the small number of samples, we focused on Asia, North America, and Oceania. Our map disclosed the highest AP richness in East Asia, South Asia, and the eastern United States of America (Figure 5b).

## 3 Discussion

Exploring the geographical distribution and driving factors of soilborne APs is an important area of interest for the One Health initiative. Here, we not only compared soilborne AP profiles in seven habitats, but also provide an unprecedented global map of soil AP distribution. We found that soilborne AP diversity was mainly driven by human activities, climatic variables, and physicochemical properties at the global scale. Specifically, agricultural land, experiencing extensive human activities, had the highest AP richness. Our global map also indicates that potential AP hotspots are located across East Asia, South Asia and eastern United States, regions that are characterized with dense populations^[^ ^21^ ^]^ and vast agricultural lands.^[^ ^22^ ^]^It is vital to increase our capacity to identify where surveillance scale‐up would be the most necessary to address future health crises driven by drug‐resistant pathogen infections.

Our study shows that agricultural land ranked the most important habitat for enriching soil AP detection. One possible reason is that APs are positively simultaneously affected by the prevalence of pathogens and the presence of ARGs, which is confirmed by agricultural soils having the highest diversity and abundance of both ARG and pathogens. Agriculture intensification, which is increasingly common for economic and social benefits,^[^ ^23^, ^24^ ^]^ reduces the biodiversity but increases the spread and enrichment of pathogens.^[^ ^25^ ^]^ Furthermore, agricultural soils often receive fertilizers from animal manures, reclaimed water irrigation, or processed sewage sludges (e.g., organic fertilizers made from wastewater treatment plant biosolids),^[^ ^26^, ^27^ ^]^ and are treated with pesticides containing some antibiotics, such as streptomycin and kasugamycin. These soil contaminants can exert selective pressures that promote the horizontal transfer of antibiotic resistome.^[^ ^4^, ^28^ ^]^

The composition of identified ARG hosts also reflected a contribution of human microbiomes to soil APs. Human pathogens were not only the most detected APs, but also the most widely distributed across all seven habitats. The most common were *Escherichia, Enterobacter, Escherichia, Mycolicibacterium*, and *Nocardia*, which are genera reported in environmental or clinically relevant studies.^[^ ^29^, ^30^, ^31^, ^32^, ^33^ ^]^ Recent research has shown that *Gammaproteobacteria*, the dominant microbial class carrying ARGs observed in Figure 2, is closely correlated with ARG abundance.^[^ ^34^ ^]^

Not only are pathogen communities driven by farming practices, the spread of ARGs is likely associated with human activities. Given the widespread use of antibiotics in humans, animals, and agriculture,^[^ ^35^ ^]^ it is no surprise that multidrug‐resistant bacteria are on the rise. The combination of antibiotic residues and ARG‐carrying and/or MGE‐carrying bacteria in agricultural soils provides important conditions for the mobilization of ARGs among microorganisms, including those considered clinical pathogens.^[^ ^36^, ^37^ ^]^ Recent studies have identified a global increase in the spread and adaptation of human‐ or healthcare‐associated multidrug resistant pathogens such as *Escherichia coli*,^[^ ^38^ ^]^ *Stenotrophomonas maltophilia*,^[^ ^39^ ^]^ *Citrobacter freundii*,^[^ ^40^ ^]^ and *Enterobacter cloacae*.^[^ ^29^ ^]^ This is further supported by the pathogen composition of the identified ARGs, which are dominated by multidrug resistance genes (Figure 3).

Another growing problem is the enrichment of the *bacA* gene due to human activities. Though *bacA* is both intrinsic and ancient in natural environments,^[^ ^41^, ^42^ ^]^ its abundance is increasing due to antibiotic abuse.^[^ ^43^, ^44^ ^]^ This gene has been detected in sediments,^[^ ^45^ ^]^ human feces,^[^ ^46^ ^]^ drinking water treatment facilities,^[^ ^47^ ^]^ activated sludge,^[^ ^48^ ^]^ and glacial and permafrost soil.^[^ ^49^, ^50^ ^]^ Recently, *bacA* was ranked as a high‐risk ARG because of its mobility and presence in ESKAPE pathogens (i.e., *Enterococcus faecium*, *Staphylococcus aureus*, *Klebsiella pneumoniae*, *Acinetobacter baumannii*, *Pseudomonas aeruginosa*, and *Enterobacter species*).^[^ ^14^ ^]^ These findings, combined with the uptrend of pathogens and ARGs with agricultural land observed in our analyses, suggest that farming practices or intense human activities are the main driving factors of AP presence in soils.^[^ ^36^, ^51^ ^]^

We generated the first atlas for soil AP richness, a necessary step to identify soil AP hotspots and to predict new sources of antibiotic resistance. Our atlas suggests that soils from the eastern United States, eastern China, and Indonesia support higher AP richness. These results are in line with findings from a global‐scale study which showed that disease severity associated with soilborne pathogens is often more significant in warmer climates.^[^ ^2^, ^52^ ^]^ As such, tropical regions are the potential hotspots for soil APs, which is consistent with surveillance and prediction results from global emerging infectious disease (EID) events as disease threat is high in these regions.^[^ ^17^ ^]^ Furthermore, temperature and precipitation appear to consistently effect AP diversity, with higher richness resulting from higher temperature and increased precipitation. One feasible explanation for such a result is that hot and wet climates are conducive to the growth and transmission of APs.^[^ ^53^ ^]^

In addition to temperature and precipitation, our global map revealed a comparatively high AP richness in New Zealand, which has large of high‐fertility grazed soils characterized by high organic carbon.^[^ ^54^ ^]^ High soil organic carbon is associated with resource availability and allows pathogens to obtain nutrients for growth and reproduction.^[^ ^55^, ^56^ ^]^ Given that New Zealand has a large dairy industry with predominantly pasture‐fed cows that are not housed, it is also suggested that intensive dairy farming leads to more APs.^[^ ^57^ ^]^

It should be acknowledged that our results might be affected by data resolution, given that the climate and other variables were obtained from global databases (although the quality and application of such data in research is rapidly increasing). It is important to note that most of our scientific resources are concentrated in economically developed areas, advocating re‐allocation of resources for efficient surveillance of predicted health risk hotspots in lower latitudes such as tropical Africa, Latin America, Africa, and Asia.^[^ ^58^ ^]^ In addition, while this study investigates the global patterns of AP richness, further studies that combine absolute abundances and diversity might be more informative to disentangle the underlying mechanisms controlling soilborne APs. Although current metagenomic sequencing data provide valuable insights into the occurrence of soil APs, a better understanding of their activity and pathogenicity in situ can be achieved through metatranscriptomics and culturomics analyses.^[^ ^59^ ^]^ Last but not least, integrating multiple surveillance platforms (from genomic to case data) will lead to a better understanding of AP emergence and spread.^[^ ^60^, ^61^ ^]^

In conclusion, our study highlights the importance of soil, particularly agricultural soils, as a potential reservoir for APs. The global distribution of AP diversity was associated with anthropogenic, climatic and soil physicochemical factors. Due to the importance of agricultural samples, a comprehensive system for surveilling AP risk in agricultural land should be a priority.^[^ ^62^ ^]^ Together, our findings advance our understanding of AP distribution in a human‐dominated world, and provide key insights to better manage the soil AP pool and understand the future potential implications of soilborne drug‐resistant pathogens for human health worldwide.

## 4 Experimental Section

### Collecting the Worldwide Dataset of Soil Metagenomes

To evaluate the current state of knowledge for soilborne APs based on metagenomic data, we collected 1385 soil metagenomic samples from NCBI Sequence Read Archive (http://www.ncbi.nlm.nih.gov/) in may 2019. The geographic information (location, latitude, and longitude) for each site and sample description (sample type) were obtained from the sample information provided by the BioSample database. These samples were divided into seven habitats (i.e., agricultural land, bare land, forest, grassland, shrubland, tundra, and wetland) according to their sample description.

### National Sampling Procedures

DNA extraction and sequencing: For this study, 258 Chinese soil samples were collected in 2018 and 2019 using a uniform sampling protocol. Briefly, the upper layer of soils or sediments (0–10 cm) was collected using a soil auger or Peterson grab sampler. All samples were refrigerated during transport using either bagged or dry ice. After transport, visible roots and stones were removed from fresh soil samples; moist soils were stored at −80 °C until DNA extraction. In all cases, DNA was extracted from soil samples using MP FastDNA SPIN Kits for soil (MP Biomedicals, Solon, OH, USA) as per the manufacturer's instructions. Equal amounts (400 mg) of soil were used to extract DNA from each sample; DNA purity and concentration were analyzed using Qubit fluorometric quantitation (Thermo Fisher Scientific, Waltham, MA, USA). Shotgun sequencing of metagenomic DNA was performed using Illumina HiSeq 4000 or Illumina 390 novaseq per150 (Illumina, San Diego, CA, USA) and produced a total of 8–37 billion paired‐end reads per sample (read length = 150 bp). Further details of all samles are described in Supporting Data 1.

### Metagenomic Quality‐Control and Assembly

In this study, raw metagenomic reads were processed by our in‐house pipelines using Trimmomatic (v2.3)^[^ ^63^ ^]^ to trim adaptors and primers, and to filter short (<50 bp) and low‐quality reads (Q20). Quality‐controlled reads were assembled per sample using MEGAHIT (v1.2.9) with a minimum contig length of 500 bp (k‐step = 10; k‐min = 27) . ^[^ ^64^ ^]^

### ARG Annotation and Abundance Calculation at Reads Level

After quality control, fastq files were aligned against the structured database SARG v2.2 to obtain the annotation of ARG profiles using ARG‐OAP (v.2.2) with default settings.^[^ ^65^ ^]^ The resulting ARG abundances were normalized by cell number. A total of 24 ARG types were identified, each representing a type of antibiotic against that the genes confer resistance. Additionally, 1204 subtypes were identified, each representing the genotypes with a collection of highly similar reference variants.^[^ ^66^ ^]^

### Pathogen Taxonomic Assignment and Relative Abundance

The candidate pathogenic genomes were based on two published reference pathogen lists (www.phi‐base.org/ and webarchive.nationalarchives.gov.uk/ukgwa/20 121 206 154 522/; www.bis.gov.uk/assets/foresight/docs/infectious‐diseases/t16.pdf), which were downloaded from NCBI RefSeq. A searchable pathogen database containing 876 pathogenic species was then constructed. The pathogen taxonomic assignment of metagenomic contigs was performed with CLARK (V1.2.6).^[^ ^67^ ^]^ For pathogen abundance, paired reads were mapped to the pathogen‐contigs with Bowtie2 v2.3.2 using the default parameters. CoverM (github.com/wwood/CoverM) was used to remove reads aligned for <90% of their length and <95% identity. Filtered bam files were passed to SAMtools to determine how many positions were covered by reads. CoverM was used to calculate the mean coverage of contigs across samples using the “trimmed_mean” mode with default parameters. The relative abundance table of pathogens was generated and normalized by the number of metagenomic reads in each sample using R script. The normalization calculation was performed according to Emerson et al.^[^ ^68^ ^]^

### ARG and Mobile Elements Identification of Pathogen‐Annotation Contigs

Contigs that were annotated as pathogenic were processed for open reading frames (ORFs) prediction. ORFs were predicted from pathogen‐annotation contigs using MetaProdigal (v2.6.3, set as ‐p meta).^[^ ^69^ ^]^ ARGs of these contigs were annotated by aligning with the deposited ones in the SARG (v2.2) database, using BLASTp with a threshold of e value ≤ 10−10, query coverage ≥80, and amino acid identity ≥80%. Then, Perl and R scripts to extract these contigs assigned to both ARGs and pathogen species (ARG‐carrying pathogens) was used. Considering the importance of mobile elements for spreading ARGs, plasmid, integrative and conjugative elements (ICEs), and integrons were detected in the ARG‐carrying pathogens. The presence of plasmid sequences was checked by PlasFlow (v1.1) with default parameters.^[^ ^70^ ^]^ The ICE‐encoding ARGs were determined based on similarity alignment (> 70%) against the ICEs database downloaded from ICEberg2.0.^[^ ^71^, ^72^ ^]^ Integron Visualization and Identification Pipeline (I‐VIP) was a well‐organized pipeline to identify, classify, annotate and visualize class 1 integrons.^[^ ^73^ ^]^ The mobility of ARGs was predicted based on either their location on the plasmid contig or co‐occurrence with an MGE (i.e, they shared a contig with an MGE).^[^ ^74^ ^]^

### Global Mapping for AP Richness in soil Environments

The ecological and environmentally relevant global layers were downloaded from www. worldclim.org, nelson.wisc.edu/sage/data‐and‐models/atlas/maps.php and neo.sci.gsfc.nasa.gov/. The anthropogenic relevant global layers were downloaded from https://datadryad.org/stash/dataset/https://doi.org/10.5061/dryad.dk1j0 and https://explorer.earthengine.google.com/#detail/CIESIN%2FGPWv4%2Funwpp‐adjusted‐population‐density (Supporting Data5). The information about layers was converted into a unified pixel grid in EPSG:4326 (WGS84) at a 0.01‐degree resolution using a nearest neighbor method. A total of 16 covariates were selected for model development. Through a comprehensive comparison on the accuracy in predictions based on their R^2^ and Root Mean Square Error, random forest was the best choice of model.^[^ ^75^, ^76^ ^]^ The prediction model was trained for the 1372 samples where AP data were available from the current project followed by global predictions of their richness for global continent. The importance of each indicator was also determined by machine learning to estimate the most critical factors influencing the soil AP diversity. Afterward, it was conducted hyperparameter tuning for random forest algorithms with optimal independent features to determine the best hyperparameter combinations. Finally, the richness value for each pixel was calculated based on a random forest model and the map was then constructed using gdal.^[^ ^77^ ^]^ It was converted all data to the Mollweide projection with a WGS84 datum to show global AP richness by ArcGIS (v10.5). To obtain the model uncertainty, it was calculated per‐pixel mean and standard deviation according to Hoogen et al.^[^ ^78^ ^]^ Using these values, the coefficient of variation (standard deviation divided by the mean predicted value) as a measure of the prediction accuracy of the model was calculated.

### Statistical Analysis

Statistical analysis and plotting were implemented in R (4.1.3). Multiple‐group comparisons were performed using Kruskal‐Walli's test. Box, violin, and histogram graphs were plotted with ggplot2. Geographic distribution maps of metagenome samples were created with the aid of R packages including rgdal and ggplot2. Taxa trees were constructed using ggtree, ggraph, ggsci, ggtreeExtra, ggstar, and treeio packages. Multiple correspondence analysis (MCA) was performed using factoextra and FactoMineR^[^ ^79^ ^]^ to investigate the correlation of the resistance profiles of APs using phylogenetic lineages. Pathogen‐ARG networks among all samples were defined by the pathogens and targeted ARGs in the same contigs. Co‐occurrence networks were built based on the presence/absence of the contigs and their encoding ARGs within APs. Network properties such as node degree were estimated by the igraph package. The final bipartite network visualizations were generated using Cytoscape 3.7.1.^[^ ^80^ ^]^ Within each habitat, a pathogens‐antibiotic types of bipartite network were visualized using circlize. Random forest models were performed with RandomForest, and accuracy in predictions were assessed based on their Root Mean Square Error and R^2^ with DALEX.

## Conflict of Interest

The authors declare no conflict of interest.

## Author Contributions

All authors contributed intellectual input to this study. The original concept and research framework was conceived by B.M. and J.X.; analysis strategies were developed by B.W. and B.M.; sample collections were coordinated by J.X., Y.W., and C.L.; data analyses were performed by B.W. with help from B.M., and Y.W.; and the manuscript was written by B.W. with the help from K.Z., E.S., Q.Y., Z.H., B.M., J.X., X.T. and D.K.

## Supporting information

Supporting Information

Supporting Information

Supporting Information

Supporting Information

Supporting Information

Supporting Information

## Data Availability

Research data are not shared.
