# Supplementary material for: Tackling Soil ARG‐Carrying Pathogens with Global‐Scale Metagenomics
Source: Adv Sci (Weinh). 2023 Jul 9;10(26):2301980. doi: 10.1002/advs.202301980 (PMC10502870; doi:10.1002/advs.202301980)
Supplement: Supplementary file 1 — Supporting Information [file ADVS-10-2301980-s002.pdf]

ADVANCED SCIENCE
Open Access

Supporting Information

for *Adv. Sci.*, DOI 10.1002/advs.202301980

Tackling Soil ARG-Carrying Pathogens with Global-Scale Metagenomics

Binhao Wang, Jianming Xu, Yiling Wang, Erinne Stirling, Kankan Zhao, Caiyu Lu, Xiangfeng Tan, Dedong Kong, Qingyun Yan, Zhili He, Yunjie Ruan and Bin Ma**

SUPPLEMENTARY INFORMATION for

Tackling Soil ARG-carrying Pathogens with Global-Scale Metagenomics

Binhao Wang, Jianming Xu, Yiling Wang, Erinne Stirling, Kankan Zhao, Caiyu Lu, Xiangfeng Tan, Dedong Kong, Qingyun Yan, Zhili He, Yunjie Ruan, Bin Ma**

J. Xu, B. Wang, Y. Wang, K. Zhao, C. Lu, B. Ma,
Zhejiang Provincial Key Laboratory of Agricultural Resources and Environment, Institute of Soil and Water Resources and Environmental Science, College of Environmental and Resource Sciences, Zhejiang University, Hangzhou 310058, China

Y. Wang, K. Zhao, C. Lu, B. Ma
Hangzhou Global Scientific and Technological Innovation Center, Zhejiang University, Hangzhou 310058, China

E. Stirling
Agriculture and Food, Commonwealth Scientific and Industrial Research Organisation, Adelaide 5064, Australia

E. Stirling
School of Biological Sciences, The University of Adelaide, Adelaide 5005, Australia

X. Tan, D. Kong
Institute of Digital Agriculture, Zhejiang Academy of Agricultural Sciences, Hangzhou, China

X. Tan, D. Kong
Xianghu Laboratory, Hangzhou, Zhejiang, 311200, China

Q. Yan, Z. He
Southern Marine Science and Engineering Guangdong Laboratory (Zhuhai), Zhuhai 519080, China

Y. Ruan
Institute of Agricultural Bio-Environmental Engineering, College of Bio-Systems Engineering and Food Science, Zhejiang University, Hangzhou 310058, China

Y. Ruan
The Rural Development Academy, Zhejiang University, Hangzhou 310058, PR China
email: jmxu@zju.edu.cn； bma@zju.edu.cn； tel: +86-13282198979, ORCID: 0000-0003-4807-4992

This PDF file includes:

Figure S1 to S8

1.SUPPLEMENTARY FIGURES

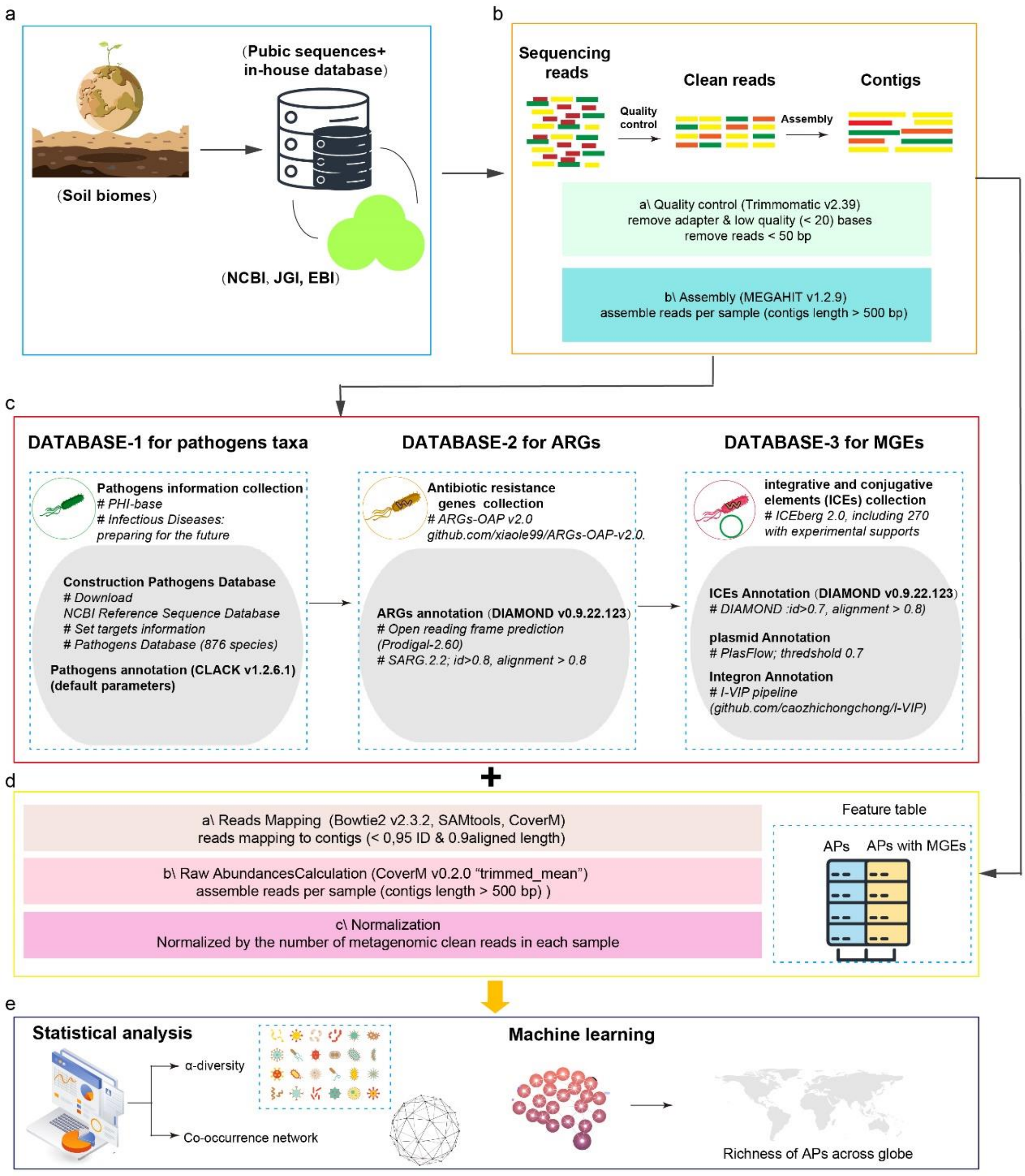

Figure S1 | Database construction workflow, data processing, and bioinformatics analyses in this study.

a, To understand the distribution of soil environmental ARG-carrying pathogens via metagenome-assembled genomes (contigs), we collected the data from 1385 (public database) and 258 (in-house) samples that had been sequenced using shotgun

metagenomics; **b,** The metagenomic sequencing data was processed by our in-house pipelines to generate high-quality reads using Trimmomatic (v2.3). The high-quality reads from each sample were individually assembled into contigs using MEGAHIT v1.2.9 with the parameters “kmers 27, 37, 47, 57, 67, 77, 87, 97, 107, 117,127, 141”; **c,** Annotation of pathogens, ARG and MGEs. Contigs were aligned against a self-constructed pathogen database using CLARK (v1.2.6). After extracting the pathogen contigs, the open reading frame was first predicted and then used on gene sequences using the recently published SARG2.0 which was implemented in ARG-OAP (v2.2). Plasmid, integrative and conjugative elements (ICEs) and integrons were detected in the ARG-carrying pathogens. The presence of plasmid sequences was checked by PlasFlow (v1.1) with default parameters. The ICE-encoding ARGs were determined based on similarity alignment (>70%) against the ICEs database downloaded from ICEberg2.0. Integron Visualization and Identification Pipeline (I-VIP) is a well-organized pipeline to identify, classify, annotate, and visualize class 1 integrons. The mobility of ARGs was predicted based on either their location on the plasmid contig or co-occurrence with an MGE (i.e. they shared a contig with an MGE gene); **d,** Paired reads were mapped to the metagenomic contigs with Bowtie2 (v2.3.2) using the default parameters. CoverM was used to remove reads aligned for <90% of their length and <95% identity. Filtered bam files were passed to SAMtools to determine how many positions were covered by reads. CoverM was used to calculate the mean coverage of contigs across samples using the “trimmed_mean‟ mode with default parameters. The relative abundance table of pathogens was generated and normalized by the number of metagenomic reads in each sample using R. The normalization calculation was performed according to Emerson et al; **e,** Using Random Forest models together with environmental factors we detected global AP richness and predicted the progression of APs under future climate.

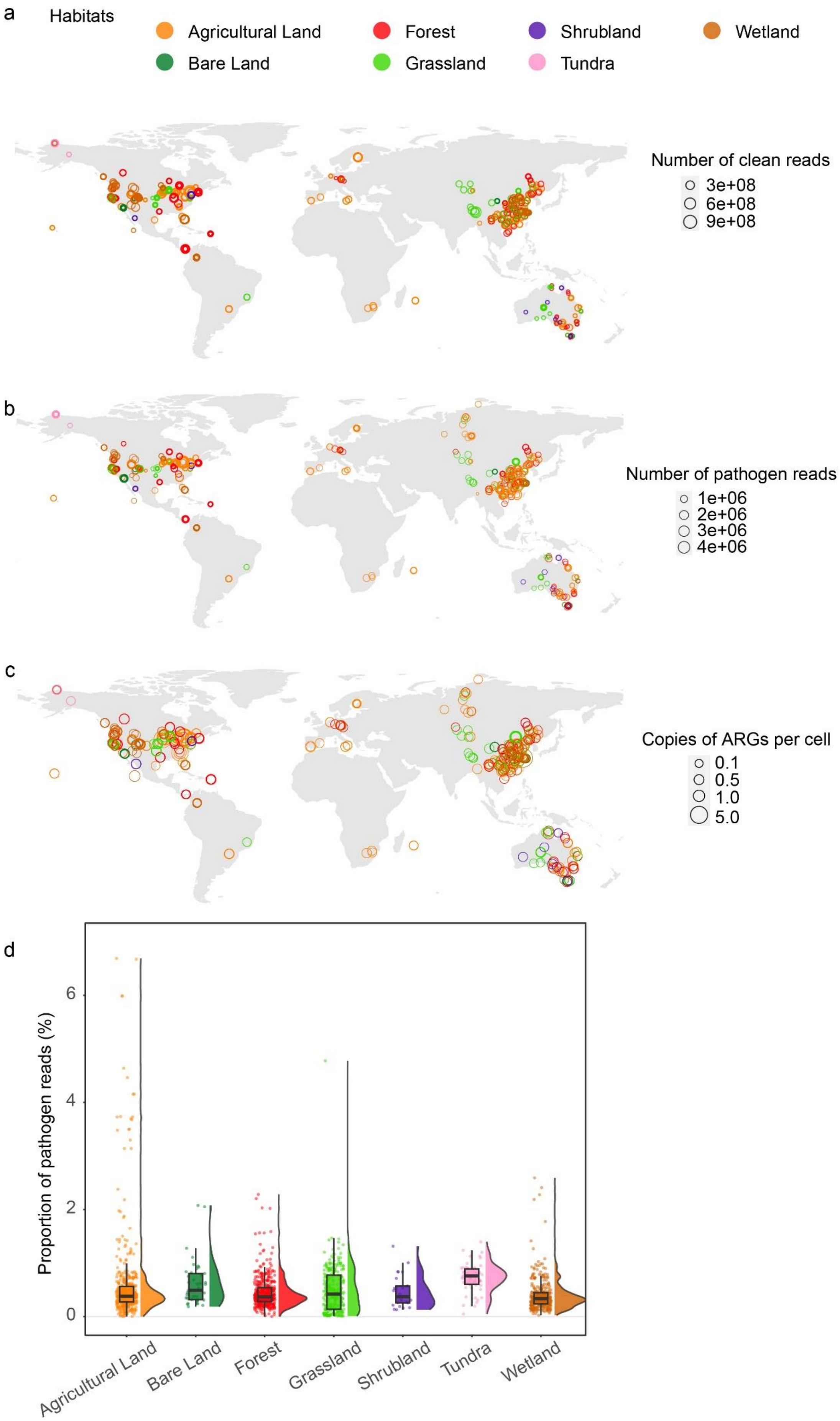

a
Habitats
Agricultural Land
Forest
Shrubland
Wetland
Bare Land
Grassland
Tundra
Number of clean reads
3e+08
6e+08
9e+08
b
Number of pathogen reads
1e+06
2e+06
3e+06
4e+06
c
Copies of ARGs per cell
0.1
0.5
1.0
5.0
d
Proportion of pathogen reads (%)
0
2
4
6
Agricultural Land
Bare Land
Forest
Grassland
Shrubland
Tundra
Wetland

Figure S2 | Overview of sample sequencing depth, pathogen and ARG abundance (n=1643).

a, Total clean reads in each sample; **b,** Pathogen reads in each sample; **c,** Copies of ARG per cell in each sample; **d,** Proportion of pathogen reads in each habitat.

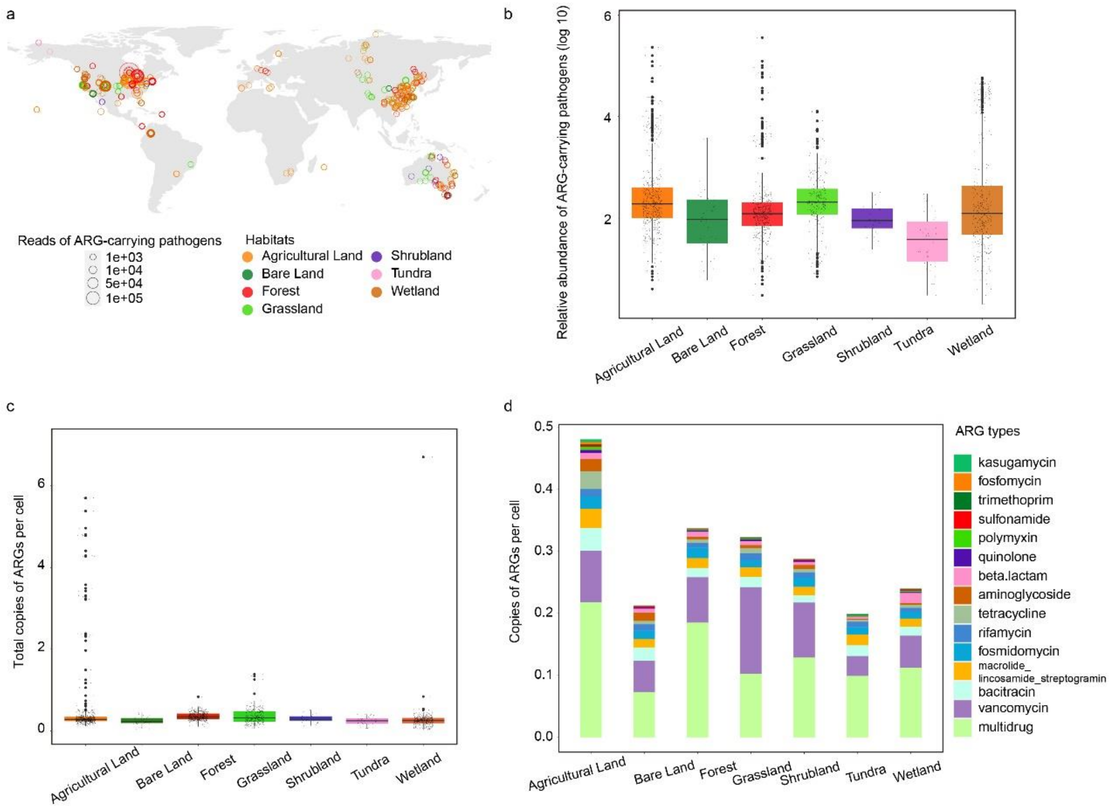

Figure S3 | Overview of abundance and ARG profiles of ARG-carrying pathogens (n=1443).

a, Reads of ARG-carrying pathogens at the global scale; **b,** Abundance of ARG-carrying pathogens in each habitat; **c,** Abundance (copies of ARG per cell) of ARGs in each habitat; **d,** Composition of antibiotic resistome in each habitat.

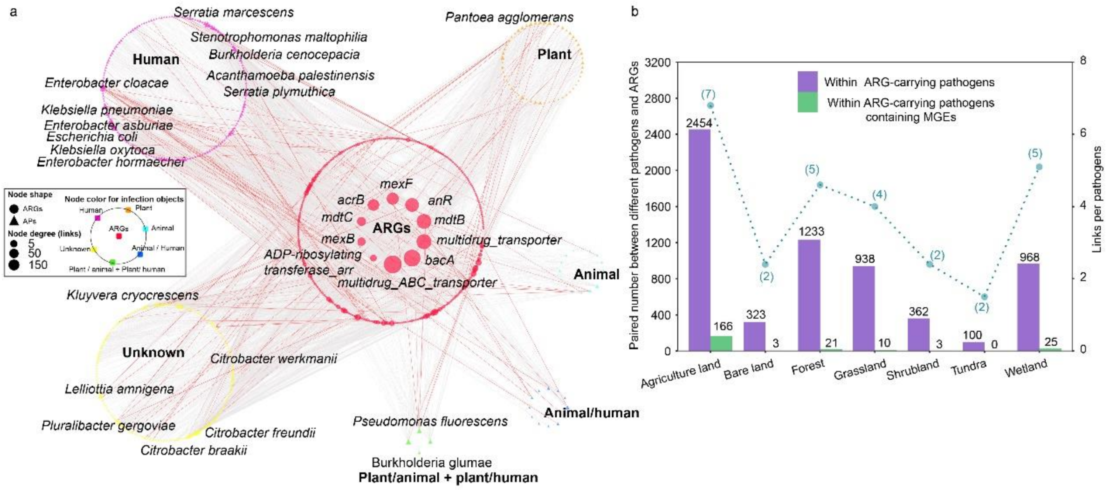

Figure S4 | Co-occurrence patterns among antibiotic resistance genes and their host pathogens across seven habitats.

a, Overall presence profile of pathogen-ARGs across all habitats based on infection objects and ARG categories. The ten ARGs and pathogens with the highest degree are listed; **b,** The coexistence characteristics of ARG-pathogens from the perspective of pairs number. Number of pairs between different pathogens and ARGs within ARG-carrying pathogens (in blue) and ARG-carrying pathogens containing MGEs (in green). Line chart shows average links per pathogen (value in parentheses)

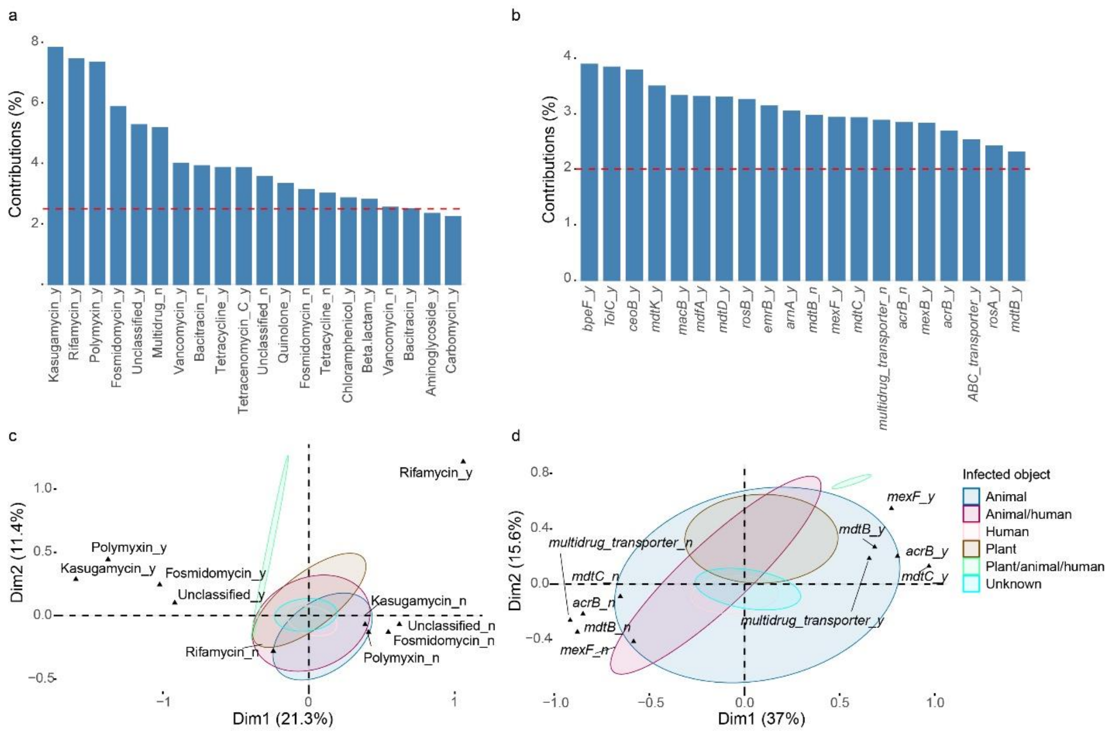

Figure S5 | Multiple correspondence analysis (MCA) of antibiotic resistance genes and their potential host pathogens.

a-b, Barplot displaying the percentage of variance explained by the respective principal component dimensions of the multiple correspondence analysis (MCA). The first four dimensions provide the percentage of variance explained by the model for 20 ARG types and 25 most linked ARG subtypes as active variables, respectively; **c-d,** Individual biplot map of the infection objects as indicated in the coloured legend under two types of active variables.

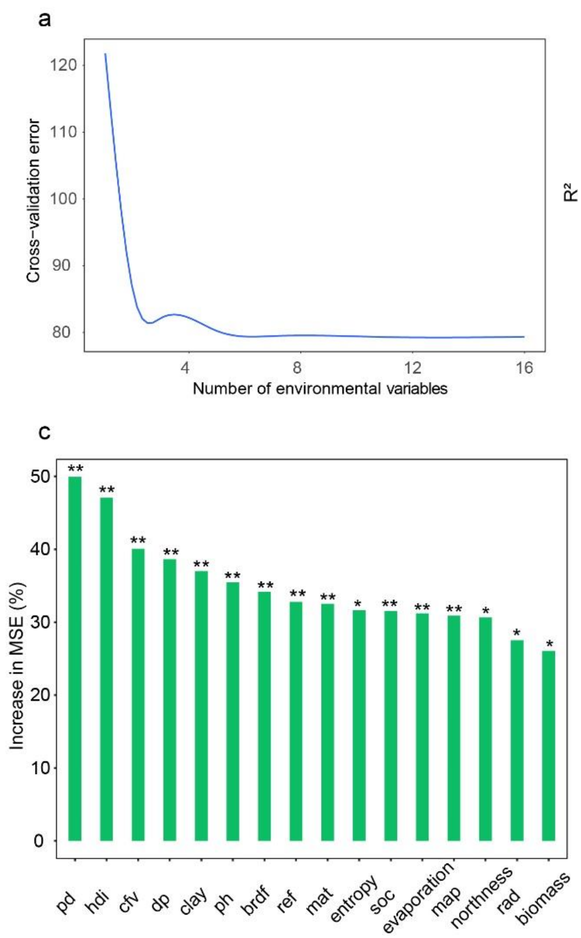

Figure S6 | Important drivers of richness and model validation.

a, Feature selection for random forest algorithms to predict AP richness based on 10-fold cross-validation; **b,** Hyperparameter tuning for random forest algorithms to predict AP richness. In the best model, ntree and mtry are 1200 and 10, respectively; **c,** Random forest mean predictor importance (percentage of increase of mean square error) of environmental indices as drivers for soil AP richness. The accuracy importance measure was computed for each tree and averaged over the forest (1000 trees). Percentage increases in the MSE (mean squared error) of variables were used to estimate the importance of these predictors, and higher MSE% values imply more important predictors. Significance codes are as follows: '**' $P < 0.01$, '*' $P < 0.05$. MSE, mean squared error. (***pd:*** Population density; ***hdi:*** Human development index; ***cfv:*** Coarse fragments volumetric; ***dp***: precipitation of driest month; ***ph****:* Soil pH; ***rad:*** BIO21-Highest_Weekly_Radiation; ***clay***: Clay content (0–2 micro meter); ***ref:*** MCD43A4.005-BRDF-Adjusted Reflectance 16-Day Global 500m; ***soc****:* soil organic carbon; ***brdf:*** Exponentially weighted difference in EVI

between adjacent pixels; ***map**:* mean annual precipitation, ***mat:*** mean annual temperature); **d,** Relationships between predicted versus observed AP richness values. R^2 values are indicated in the upper left corner, solid diagonal lines indicates a 1:1 relationship between predicted and observed points.

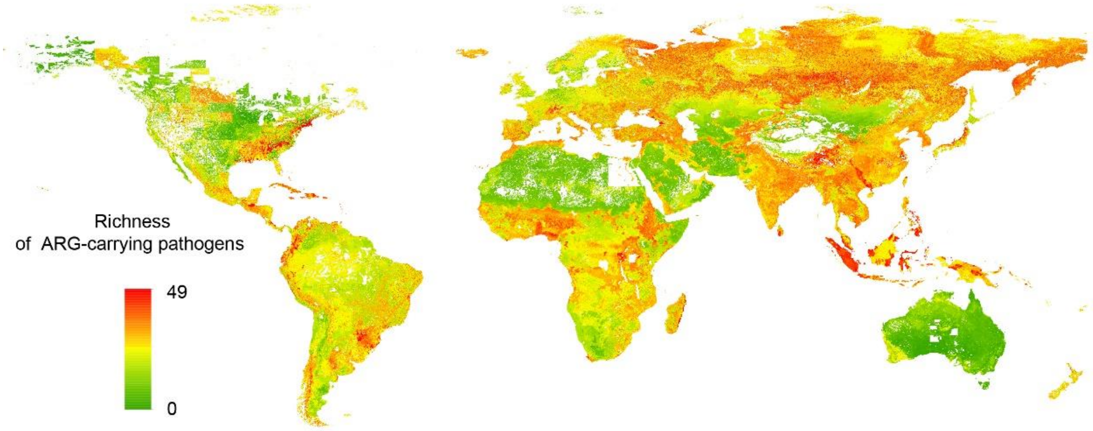

Figure S7 | Global map of soil-borne ARG-carrying pathogen (AP) richness.

AP richness with prediction results derived from random forest analysis. Map was drawn in ArcGIS at 0.01 degree resolution using projection WGS84.

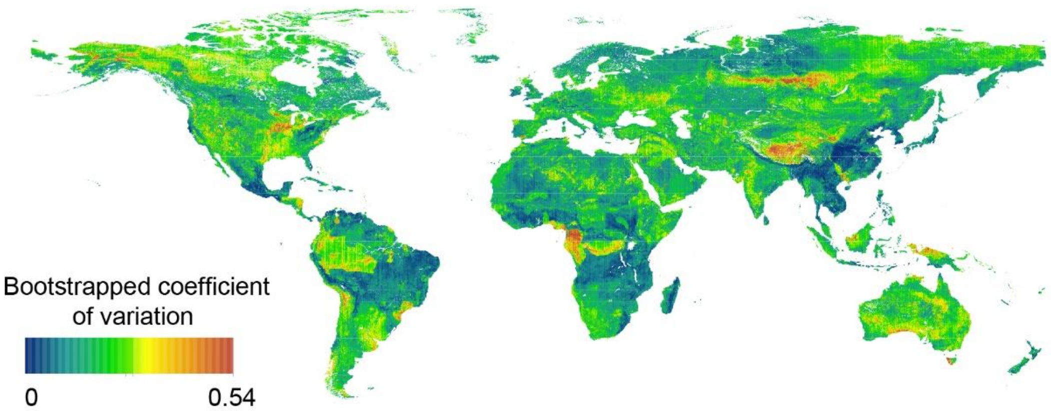

Figure S8 | Model accuracy assessment across the globe.

Bootstrapped (100 iterations) coefficient of variation (standard deviation divided by the mean predicted value) as a measure of prediction accuracy. Sampling was stratified by habitats.

References:

Bolger AM, Lohse M, Usadel B. Trimmomatic: a flexible trimmer for Illumina sequence data. Bioinformatics 30, 2114-2120 (2014).

Li D, Liu C-M, Luo R, Sadakane K, Lam T-W. MEGAHIT: an ultra-fast single-node solution for large and complex metagenomics assembly via succinct de Bruijn graph. Bioinformatics 31, 1674-1676 (2015).

Yin X, et al. ARGs-OAP v2. 0 with an expanded SARG database and Hidden Markov Models for enhancement characterization and quantification of antibiotic resistance genes in environmental metagenomes. Bioinformatics 34, 2263-2270 (2018).

Yin X, et al. An assessment of resistome and mobilome in wastewater treatment plants through temporal and spatial metagenomic analysis. Water Res 209, 117885 (2022).

Ounit R, Wanamaker S, Close TJ, Lonardi S. CLARK: fast and accurate classification of metagenomic and genomic sequences using discriminative k-mers. BMC genomics 16, 1-13 (2015).

Emerson JB, et al. Host-linked soil viral ecology along a permafrost thaw gradient. Nat Microbiol 3, 870-880 (2018).

Hyatt D, LoCascio PF, Hauser LJ, Uberbacher EC. Gene and translation initiation site prediction in metagenomic sequences. Bioinformatics 28, 2223-2230 (2012).

Krawczyk PS, Lipinski L, Dziembowski A. PlasFlow: predicting plasmid sequences in metagenomic data using genome signatures. Nucleic Acids Res 46, e35-e35 (2018).

Sabino YNV, et al. Characterization of antibiotic resistance genes in the species of the rumen microbiota. Nat Commun 10, 1-11 (2019).

Liu M, et al. ICEberg 2.0: an updated database of bacterial integrative and conjugative elements. Nucleic Acids Res 47, D660-D665 (2019).

Zhang AN, Li L-G, Ma L, Gillings MR, Tiedje JM, Zhang T. Conserved phylogenetic distribution and limited antibiotic resistance of class 1 integrons revealed by assessing the bacterial genome and plasmid collection. Microbiome 6, 1-14 (2018).

Sun J, et al. Environmental remodeling of human gut microbiota and antibiotic resistome in livestock farms. Nat Commun 11, 1-11 (2020).
